# Pulsed Microfluid Force-Based On-Chip Modular Fabrication for Liver Lobule-Like 3D Cellular Models

**DOI:** 10.34133/2021/9871396

**Published:** 2021-04-08

**Authors:** J. Cui, H. P. Wang, Q. Shi, T. Sun

**Affiliations:** ^1^Science and Technology on Electronic Test and Measurement Laboratory, North University of China, Taiyuan 030051, China; ^2^Intelligent Robotics Institute, School of Mechatronical Engineering, Beijing Institute of Technology, Beijing 100081, China; ^3^Beijing Advanced Innovation Center for Intelligent Robots and Systems, Beijing Institute of Technology, Beijing 100081, China; ^4^Key Laboratory of Biomimetic Robots and Systems (Beijing Institute of Technology), Ministry of Education, Beijing 100081, China

## Abstract

In vitro three-dimensional (3D) cellular models with native tissue-like architectures and functions have potential as alternatives to human tissues in regenerative medicine and drug discovery. However, it is difficult to replicate liver constructs that mimic in vivo microenvironments using current approaches in tissue engineering because of the vessel-embedded 3D structure and complex cell distribution of the liver. This paper reports a pulsed microflow-based on-chip 3D assembly method to construct 3D liver lobule-like models that replicate the spatial structure and functions of the liver lobule. The heterogeneous cell-laden assembly units with hierarchical cell distribution are fabricated through multistep photopatterning of different cell-laden hydrogels. Through fluid force interaction by pulsed microflow, the hierarchical assembly units are driven to a stack, layer by layer, and thus spatially assemble into 3D cellular models in the closed liquid chamber of the assembly chip. The 3D models with liver lobule-like hexagonal morphology and radial cell distribution allow the dynamic perfusion culture to maintain high cell viability and functional expression during long-term culture in vitro. These results demonstrate that the fabricated 3D liver lobule-like models are promising for drug testing and the study of individual diagnoses and treatments.

## 1. Introduction

The liver is an essential organ that maintains life activities such as protein synthesis, vitamin storage, and detoxification [1]. However, liver disease is a common disease with a high incidence rate and high risk [2]. To explore the mechanism of liver disease for prevention and personalized diagnosis, a predictive platform or model that can reproduce in vivo bioactivities and the metabolism process of hepatocytes is needed. In vitro liver models have attracted attention in tissue engineering and biomedicine because they can involve autologous hepatocytes in an engineered extracellular matrix (ECM). Although various engineering approaches have been introduced to fabricate tissue-like cellular models, the construction of liver-mimetic tissues remains a major challenge as native liver tissues are highly complex 3D architectures with hierarchical cell distributions and vascular networks. It has been demonstrated that the cell function expression is affected by its surrounding environment, including ECM and neighbor cells [3–5]. Therefore, a suitable 3D microenvironment that physiologically mimics native tissue is a basic and crucial step in recreating liver models for the repair and restoration of tissue functions.

Physiological liver functions, such as drug metabolism, largely depend on the transport and diffusion of blood vessels [6]. Owing to the precious control of microfluidics to a microscaled fluid, microfluidic-based culture platforms and microbioreactors have been developed for continuous perfusion cultures of cellular tissues, which are expected to promote cell mass transfer and maintain cell activity [7, 8]. However, most of the cellular tissues in these platforms are cell spheroids or clusters with simple spatial configurations that cannot mimic the complex 3D structure of human liver tissues. The liver is composed of repeating basic structural and functional units called liver lobules [9]. In the liver lobule, various types of cells are radially arranged around the central vein and organized into hierarchical heterogeneous microtissues with an outline of a six-sided cylinder. To fabricate a liver model for in vitro culture, a 3D architecture with hierarchical cell distribution is required to maintain cell survival and normal cell phenotype [10]. Additionally, an embedded vascular structure is indispensable for sufficient mass transfer between cells and nutrients because the cellular diffusion of oxygen and nutrients is limited to 200 *μ*m [11, 12].

To fabricate 3D scaffolds and substrates for cell culture, various hydrogels have been developed to mimic in vivo ECM [13, 14]. Although some hydrogels have appropriate mechanical properties, it is still challenging to manipulate the microscale hydrogel models without damage to the structure or embedded cell viability. Microfluidics allow for manipulating cells and other microscale objects in the customized microchannel by controlling the velocity and direction of the microflow. For example, Yue et al. designed a three-layered microfluidic channel that can assemble the circular micromodules layer by layer to form tube-like integration [15]. Chung et al. developed a railed microfluidic channel with a bump embedded at the bottom of the channel. The micromodules moving horizontally can be rotated by 90° at the bump and stacked vertically at the end of the channel [16]. However, these microchannels are not versatile enough to assemble arbitrarily shaped micromodules because of the restriction of the predesigned microfluidic channel. Recent advanced bioprinting approaches enable multilayer printing of complex 3D tissue constructs by introducing coaxial nozzles or channels [17–19]. However, the nozzle needs to be redesigned according to the customized hierarchical shapes [20]. Additionally, high printing resolution requires nozzles with small diameters, which can damage cells by shear stress during printing [21, 22]. Bottom-up methods, or modular assembly methods, which fabricate 3D cellular microstructures by assembling cell-laden building blocks, have the potential to achieve tissue-like, 3D complex microarchitectures by controlling the features of individual building blocks [23, 24]. To avoid structure deformation and cell damage during the assembly process, dielectrophoresis, magnetic force, optical force, and other field force have been utilized for noncontact control and the assembly of heterogeneous cell units [25–27]. However, complex peripheral equipment is required, and only limited spatial control can be realized. Considering the complex architecture and function of liver tissue, a flexible and efficient noncontact 3D assembly method is needed for fabricating liver lobule-like hierarchical constructs with perfusion ability and liver functions.

**Figure 1:**
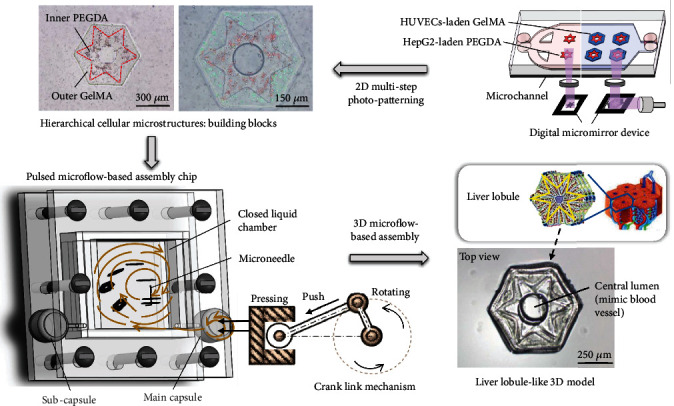
Schematic of the pulsed microflow-based on-chip assembly for the construction of perfusable liver lobule-like 3D models. By pressing the main capsule through the crank link mechanism, a circular microflow can be formed in the chamber, which can drive the microstructures to stack on the microneedle.

In this study, we developed a pulsed microfluid force-based on-chip 3D assembly method that allows for noncontact assembly of hierarchical cellular microstructures into perfusable 3D cellular models with liver lobule-like architecture and radial cell distribution, as shown in Figure 1. The cellular microstructures with hierarchical features were produced through multistep photopatterning of different hydrogels with different types of cells. To assemble the microstructures into a 3D cellular integration, a pressure-assistant chip with a closed liquid chamber was designed and optimized to generate pulsed microflow for noncontact assembly of the microstructures into 3D constructs with a central lumen. The resultant 3D models have a liver lobule-like hexagonal morphology and hierarchical cell distribution but also allow for a dynamic perfusion culture to maintain high cell viability and functional expression during long-term culture in vitro. Experimental results demonstrated that the proposed method is capable of fabricating 3D cellular models with liver lobule-like architecture and biological significance, which can potentially be used in pharmacological and pathological research.

## 2. Materials and Methods

### 2.1. Experimental Design

To construct the liver lobule-like 3D cellular model through modular assembly, microstructures with liver lobule-like morphology and hierarchical cell distribution should be prepared before experiments. The cell-laden microstructures as assembly units were fabricated using a previously reported digital on-chip UV exposure system [28]. Using in situ multistep photopatterning, different cell-laden hydrogels were sequentially injected into the microchannel and photocrosslinked into nested structures in the same position by controlling the digital micromirror device. Therefore, hierarchical microstructures encapsulating different types of cells in different substructures are formed. To mimic the liver lobule, a hierarchical microstructure integrating an outer hexagonal pattern with a diameter of 1 mm and an inner radial pattern was produced. A central hole with a diameter of 300 *μ*m was designed in the microstructure, which could form an embedded lumen after stacking to mimic the central vein of the liver lobule.

The device for noncontact assembly of the microstructures consisted of a pulsed microflow-based assembly chip and a crank link system. The pulsed microflow chip possesses a closed liquid chamber fabricated by a polydimethylsiloxane (PDMS) block with a square cavity and PDMS substrate. The PDMS cavity was 15 mm × 15 mm × 1.2 mm. To avoid liquid leakage during assembly from the chamber, two polymethyl methacrylate (PMMA) plates were used to clamp and seal the PDMS cavity and substrate. However, direct clamping of PMMA plates on PDMS can compress the elastic PDMS as well as the liquid chamber, which may influence the movement of microstructures in the chamber. Therefore, the middle of each plate was designed with a square hole slightly larger than the chamber to clamp and compress the edge of the chamber. A microneedle with a diameter of 200 *μ*m was fixed in the chamber for the collection of the microstructures. Two holes with a diameter of 1.2 mm were punched on the relative position of the chamber sides. Two droppers serving as compressible water capsules were installed on the chamber through the holes. The one dropper near the microneedle was defined as the main capsule. The other dropper, the subcapsule, was used to balance the pressure of the chamber. By pressing the main capsule through the crank link mechanism, a circular microflow was formed in the chamber, which can drive the microstructures to assemble in the chip. Similarly, by pressing the subcapsule, circular microflow with the opposite direction can be formed, which can drive the microstructure to move in the opposite direction.

### 2.2. Simulation Analysis

To analyze the formation of the pulsed microflow and optimize the assembly parameters, simulation of the pulsed microflow in the chamber was performed using COMSOL software. The simulation model was designed by SolidWorks and imported into COMSOL. The model was defined on the *X*–*Z* plane. A displacement along the *Y*-axis was applied to the capsule to generate pressure in the liquid chamber. The liquid chamber was considered inelastic, and the laminar flow was considered incompressible.

### 2.3. Cell Culture

Human hepatoma cells (HepG2) and human umbilical vein endothelial cells (HUVECs) were purchased from ATCC, USA. HepG2 cells were cultured with DMEM (Dulbecco's modified Eagle medium, Solarbio, China) containing 10% FBS (Fetal Bovine Serum, GIBCO, USA) and 1% penicillin-streptomycin (Solarbio) at 37°C under 5% CO_2_. HUVECs were cultured with an endothelial culture medium (ScienCell, USA). When the cell confluence area reached 80% in the culture dish, cells were detached from the dish using trypsin (Solarbio) and blended with hydrogel solutions before photopatterning.

### 2.4. Materials

Poly(ethylene glycol) diacrylate (PEGDA) and gelatin methacrylate (GelMA) were used to fabricate the inner cell-laden pattern and outer cell-laden pattern of the hierarchical microstructure. PEGDA (Mw = 3400 Da) was purchased from Laysan Bio. Inc. (USA). 2-Hydroxy-1-(4-(hydroxyethoxy)phenyl)−2-methyl-1-propanone (Irgacure 2959) as a photoinitiator (PI) was purchased from BASFSE (Germany). For cell culture, PEGDA was modified with RGDS peptides (Sigma-Aldrich, USA) [29]. A PEGDA solution with 20% (*w*/*v*) PEGDA, 0.5% (*w*/*v*) PI, 5 mM RGDS-modified PEGDA, and 1 × 10^6^ mL^−1^ HepG2 cells in DMDM (HYCLONE, USA) was used to fabricate the inner radial pattern with cell encapsulation through photopatterning.

GelMA was prepared by synthesizing 10% (*w*/*v*) gelatin and 20% (*w*/*v*) methacrylic anhydride according to a previous report [30]. Lithium phenyl-2,4,6-trimethyl-benzoylphosphinate (LAP) was purchased from Allevi, USA. To obtain the GelMA solution, 10% (*w*/*v*) GelMA, 0.5% (*w*/*v*) LAP, and DMEM with 1 × 10^6^ mL^−1^ HUVECs were mixed at 37°C.

### 2.5. Quantification of the Cell Viability and Function

Cell-laden microstructures and assembled 3D cellular models were cultured in a Petri dish coated with PDMS at 37°C under 5% CO_2_. PDMS prevented encapsulated cells from adhering to the dish. The culture medium was changed every 2 days. The cell viability was quantified by a CCK-8 kit. Live and dead cells were stained with calcein AM and propidium iodide stains (Molecular Probes, USA), and the fluorescent images were analyzed using ImageJ software.

For perfusion culture, two micropipettes with pulled tips were used as the inlet and outlet of the perfusion model. They were sealed to the 3D cellular model by photocrosslinking of PEGDA at the connecting position. The perfusion culture medium with a velocity of 50 *μ*L/min was injected into the 3D model and reserved at the outlet. For scale-up, a micropillar array with a PDMS substrate and copper wire was designed.

For evaluation of the liver function of the hierarchical 3D models, the culture medium of the 3D cellular models was collected every 12 hours and stored at −80°C before use. Urea synthesis was assessed using a human urea assay kit (Sigma) according to the manufacturer's instructions, and the value was normalized to the cell number.

### 2.6. Statistical Analysis

All values were represented as the mean ± standard deviation (SD). All experiments were performed with *n* = 3 or greater.

## 3. Results

### 3.1. On-Chip 3D Assembly Principles

**Figure 2:**
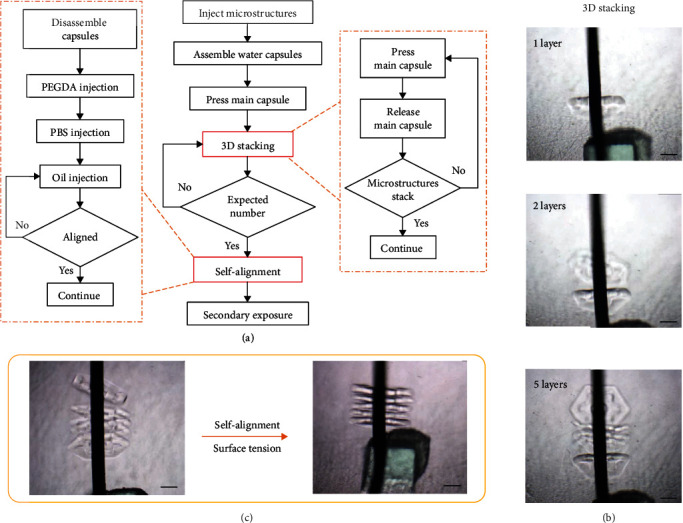
On-chip 3D assembly of the hierarchical microstructures: (a) flow chart of the whole 3D assembly process; (b) 3D stacking through pulsed microfluid force interaction; (c) self-alignment through the surface tension of the hydrophilic-hydrophobic interaction. Scale bars: 300 *μ*m.

The 3D assembly of the cell-laden microstructures based on the pulsed microflow chip includes two steps, 3D stacking and self-alignment of the microstructures, as shown in Figure 2(a). Before assembly, the microstructures with culture medium were injected into the chamber of the assembly chip through the holes used for installing the water capsules. The capsules were then inserted into the holes, and the chamber was sealed. The chip was fixed vertically to deposit the microstructures on the bottom of the liquid chamber. The link mechanism was then used to press the main capsule with a preset frequency. When the main capsule was pressed, liquid in the main capsule trended to squeeze into the chamber so that microflow was formed. The microflow extruded from the tip of the main capsule, flowing upward and then downward, creating circulation in the chamber in response to the chamber structure. Driven by the circular microflow, the microstructures at the bottom of the chamber first moved upward and then fell to the bottom. During the falling process, the microstructures were collected on the microneedle once the hole of the microstructure was passed through the microneedle (Figure 2(b)). By repeatedly pressing the main capsule to generate regular pulsed microflow, the microstructures were stacked layer by layer on the microneedle using the flexible and dynamic interaction between the circular microflow and microstructures.

After the 3D stacking of the microstructures, posture alignment and integration were needed because the microstructures on the microneedle were irregular and discrete. To avoid damage to the microstructures, on-chip self-alignment was proposed based on hydrophilic-hydrophobic interactions. First, the water capsules were removed, and the PEGDA solution was injected into the chamber to cover the surface of the stacked microstructures with a PEGDA prepolymer. The phosphate buffer solution (PBS, Solarbio) was injected to wash out the extra PEGDA solution on the microstructures. Mineral oil was then injected into the chamber, surrounding the microstructures. Because the surface of the microstructures is hydrophilic and the mineral oil is hydrophobic, surface tension was generated on the surface of the microstructures, which can drive the microstructures to minimize their exposure surface in the oil. Driven by the surface tension, the microstructures on the microneedle were simultaneously translated and rotated to form an aligned regular morphology (Figure 2(c)). Finally, the aligned microstructures were integrated by photocrosslinking the PEGDA prepolymer on the surface of the microstructures using 10 seconds of UV exposure. Therefore, a 3D construct with regular tissue-like architecture and a vessel-like central lumen was obtained.

### 3.2. Optimization of the Chip Assembly

**Figure 3:**
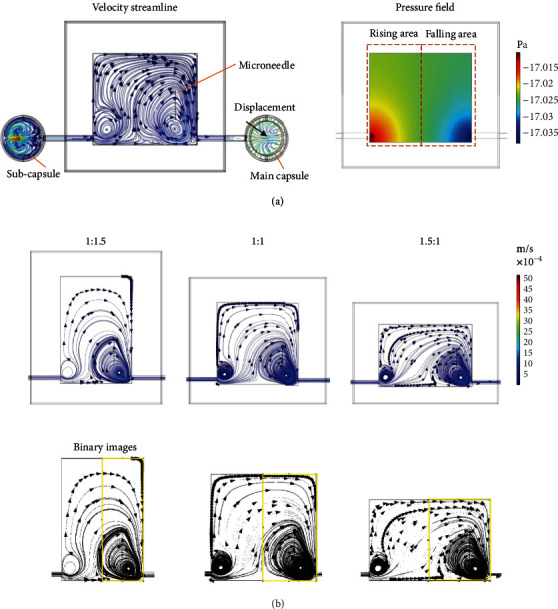
Simulation of the circulating microflow in the liquid chamber by increasing and decreasing the displacement of the main capsule: (a) simulation of the velocity streamline and pressure in the liquid chamber; (b) simulation of the streamline distribution in the liquid chamber with different aspect ratios.

The circular microflow formation in the chamber was simulated using COMSOL software. As shown in Figure 3(a), by increasing and decreasing the displacement of the main capsule, circular flow was generated in the chamber. Liquid flow from the bottom to the top of the microneedle can drive microstructures to move up and down and be collected on the microneedle. Although some microstructures may not be collected on the microneedle in one cycle of flow, the fluid at the bottom of the right half can quickly drive these microstructures up and down again until they are all collected on the microneedle. The liquid flow from the bottom to the top of the chamber roughly divides the microstructure movement area into left and right parts. Accordingly, the chamber was divided into two parts: the rising area and the falling area. The microneedle was fixed in the falling area for the collection of the microstructures. The microflow with a higher velocity at the beginning drove the microstructures to quickly rise, which determined the number of driven microstructures. The downward microflow with lower velocity could prevent damage of the microstructures by the microneedle. The microflow in the rising area determined the number of driven microstructures, and the microflow in the falling area influenced the stacking efficiency on the microneedle.

As the circular microflow is attributed to the restriction of the structure of the chamber, the structure size of the chamber can influence the formation of the circular microflow, which is related to the assembly efficiency. High assembly efficiency is essential to maintain cell viability during in vitro experiments. To optimize the chamber and improve the efficiency of the 3D stacking, a fluid simulation of the chip models with the same volumes and different aspect ratios of the liquid chamber was performed. The liquid chambers with aspect ratios of 1 : 1.5, 1 : 1, and 1.5 : 1 were designed and simulated with the same displacement of the main capsule. The flow simulation of streamline distributions at the maximum displacement of the main capsules is shown in Figure 3(b). The velocity streamline is denser in the falling area (dotted box), which means the microstructure movement is more influenced by the circular flow in the falling area. The higher density of the streamline, the higher the driving force and the faster the circulation of the microstructure movement, which improves the assembly efficiency. The simulation results were binarized and analyzed using ImageJ software to quantify the distribution area of the streamline in the falling area.  Table 1 demonstrates the proportions of the streamline distribution area relative to the falling area in the different models. With the aspect ratio of 1 : 1, the maximum distribution of the circular microflow in the falling area was achieved, which was more conducive to the 3D assembly of the microstructures. Therefore, the chamber with an aspect ratio of 1 : 1 was adopted for noncontact 3D assembly of the liver lobule-like microstructures.

### 3.3. Effect of Pulse Frequency on the Assembly Efficiency

**Figure 4:**
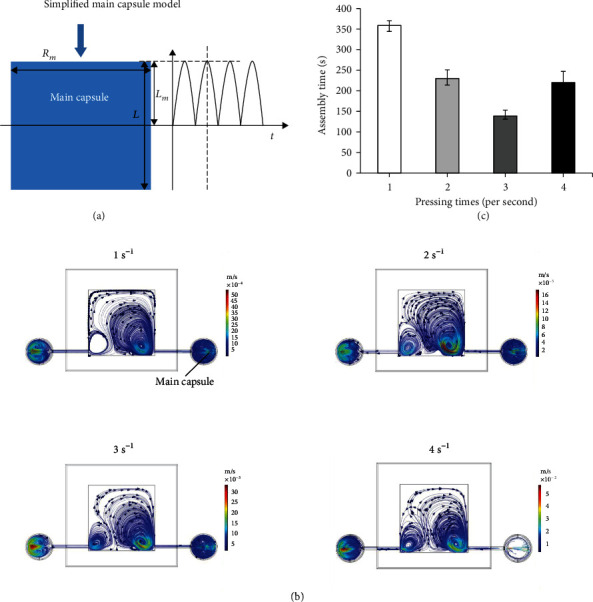
The effect of the pressing frequency of the main capsule on assembly efficiency: (a) the pressing geometry of the simplified main capsule model; (b) simulation of the microflow with different pressing frequencies of the main capsule; (c) experimental time of assembling 10 microstructures under different pressing frequencies.

With regular pulsed circular microflow, the microstructures can be driven to stack layer by layer on the microneedle. Therefore, the pressing frequency of the main capsule for generating the pulsed microflow greatly affects the success rate of the 3D stacking. To analyze the effect of the pressing frequency on the microflow, the main capsule was simplified as a compressible cylinder with a fixed radial diameter *R*_*m*_ and variable length of *L*_*m*_ = 2/*L* to *L*_*m*_ = 0. The geometry of the simplified model is shown in Figure 4(a). The pressing displacement *D*_height_ of the main capsule by the linkage mechanism can be expressed by

(1) $$\begin{array}{c} D_{\text{height}}=A\left|\sin\left(\omega t+\varphi_{0}\right)\right|, \end{array}$$

where *A* is the pressing amplitude and *A* = *L*_*m*_ and *φ*_0_ is the initial displacement of the mechanism. Considering *φ*_0_ = 0, the pressing frequency *f* can be expressed as

(2) $$\begin{array}{c} f=\frac{\omega }{2\pi }. \end{array}$$

Therefore, Equation (1) can be expressed as

(3) $$\begin{array}{c} D_{\text{height}}=L_{m}\left|\sin\left(2\pi ft\right)\right|. \end{array}$$

According to the displacement change of the water capsule in Figure 4(a) and Equation (3), the pressing speed *v*_press_ can be described as

(4) $$\begin{array}{c} v_{\text{press}}=\frac{{dD}_{\text{height}}}{dt}=A\omega \cos\left(\omega t+\varphi_{0}\right)=2\pi L_{m}f\cos\left(2\pi ft\right). \end{array}$$

Therefore, the volume of water *V*_water_ extruded from the capsule per unit time can be calculated by

(5) $$\begin{array}{c} V_{\text{water}}=\frac{\pi R_{m}^{2}v_{\text{press}}}{4}. \end{array}$$

Combining Equations (5) and (4),

(6) $$\begin{array}{c} V_{\text{water}}=\frac{\pi^{2}R_{m}^{2}L_{m}f\cos\left(2\pi ft\right)}{2}. \end{array}$$

Considering the diameter of the outlet of the water capsule as *R*_0_, the initial velocity of the fluid extruded from the water capsule can be calculated as

(7) $$\begin{array}{c} v_{0}=\frac{2\pi^{2}R_{m}^{2}L_{m}f\cos\left(2\pi ft\right)}{\pi R_{o}^{2}}. \end{array}$$

In Equation (7), the initial velocity *v*_0_ of the circular microflow is related to the pressing frequency *f* of the main capsule. Higher frequency leads to greater initial velocity and driven force on the microstructures.

The fluid simulation of the microflow generated by different pressing frequencies was performed. As shown in Figure 4(b), comparing the simulated microflow at the maximum displacement, the higher the pressing frequency, the larger the velocity of the microflow. The simulation result is consistent with the theoretical trajectory, which validates the pressing model of the water capsule. In the actual experiment, the pressing frequency of 1, 2, 3, and 4 times per second was set. Ten numbers of the microstructures were used as the sample for assembly tests under different frequencies. Each test was replicated five times, and each assembly time was recorded. As shown in Figure 4(c), with the increase in the pressing frequency, the assembly time was decreased and the assembly efficiency was improved. However, the assembly time at a frequency of 4 times per second was longer than that of 3 times per second because the fluid force at the frequency of 4 times per second was too large, which can damage the microstructures. Therefore, the pressing frequency of the main capsule for assembling liver lobule-like 3D models was set to 3 times per second.

### 3.4. Long-Term Coculture of a Liver Lobule-Like 3D Model

**Figure 5:**
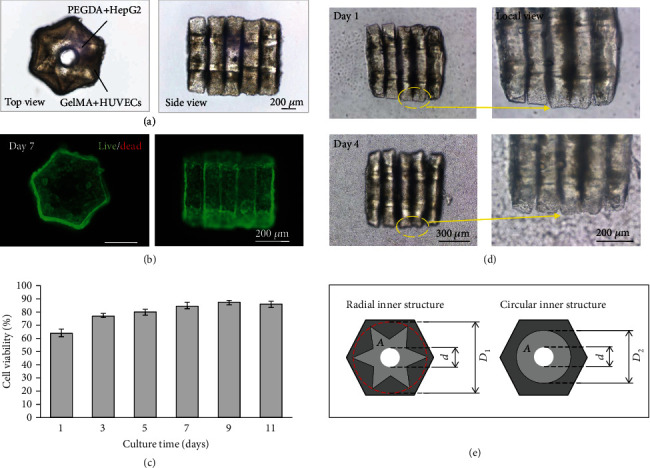
Long-term coculture of the liver lobule-like 3D model. (a) Bright-field image of the liver lobule-like 3D model consists of an inner HepG2-laden PEGDA substructure and outer HUVEC-laden GelMA substructure. (b) Live/dead staining of the cells in the 3D model. (c) Evaluation of the cell viability of the liver lobule-like 3D model during in vitro culture using CCK-8. (d) Cell proliferation in the 3D model during in vitro culture. (e) Geometries of the cross-section of the liver lobule-like 3D model and a comparative example of a multilayered 3D with a traditional circular inner structure.

Based on the optimized values of the key parameters, the hierarchical cell-laden microstructures were assembled into 3D liver lobule-like models with hierarchical cell distribution and a central lumen using the pulsed microflow-based on-chip spatial assembly method. The assembled cellular 3D models were then cultured in a Petri dish for several days. As shown in Figure 5(a), the 3D liver lobule-like model consisted of an inner HepG2-laden PEGDA substructure with a radial-like pattern and the outer HUVEC-laden GelMA substructure with a hexagonal pattern to mimic the liver lobule with hexagonal morphology and radial cell distribution. A longitudinal lumen was formed in the center of the model to mimic the central vein of the liver lobule and reduce the distance between the encapsulated cells and nutrients. Although GelMA has good cell compatibility and biodegradability for cell culture in vitro, it is fragile and easy to deform during 3D assembly, and thus tough to construct a robust heterogeneous 3D microarchitecture for perfusion. Therefore, PEGDA with better mechanical properties (with approximately 3-fold greater elastic modulus than GelMA) was involved in the 3D model as a skeleton to support GelMA and achieve hierarchical distribution of different types of cells.

The cells maintained high cell viability after long-term coculture of the 3D cellular model (Figures 5(b) and 5(c)). High cell viability demonstrated that the proposed method enabled the construction of heterogeneous cell-laden 3D models for in vitro cell coculture. In Figure 5(d), cells spread and connected with neighboring cells and appear to be at a higher density after culture, most likely indicating increased proliferation. Moreover, cell spreading along the hexagonal morphology is important to maintaining liver lobule-like morphology.

The radial pattern in the liver lobule-like model is essential as it mimics the architecture of the liver lobule and increases the contact area for interactions of HepG2 cells and HUVECs. Additionally, the design of the inner radial substructure can enhance the stability of the 3D architecture. Considering the hierarchical 3D model as a tube-like structure, its stability is inversely proportional to the slenderness ratio *λ*, which can be described as follows:

(8) $$\begin{array}{c} \lambda =\frac{\mu \cdot l}{i}, \end{array}$$

where *μ* is the length factor, determined by the constraints on the structure, *l* is the length of the structure, and *i* is given by the following equation:

(9) $$\begin{array}{c} i=\sqrt{\frac{I}{A}}=\sqrt{\frac{\left(\pi D^{4}/64\right)\left(1-d^{4}/D^{4}\right)}{A}}, \end{array}$$

where *A* is the cross-sectional area, *I* is the section moment of inertia, and *D* and *d* are the diameters of the cross-section and central lumen, respectively. Compared with a multilayered structure that has a traditional circular inner structure with the same cross-sectional area *A*, the diameter of the radial pattern *D*_1_ is larger than that of the traditional circular pattern *D*_2_, such that *I* of the radial pattern is larger than that of the circular pattern (Figure 5(e)). Therefore, *λ* of the radial structure is smaller than that of the circular structure under the same constraints, which indicates that the inner radial substructure is more stable for supporting the 3D architecture.

### 3.5. Evaluation of the Liver Lobule-Like Model

For an actual liver, cell survival and function expression are largely dependent on the diffusion and permeation of blood vessels. To promote cell activity during in vitro culture, a perfusion system based on the lumen of the 3D liver lobule-like model was established for the dynamic culture of the 3D model. A syringe pump was connected to one side of the central lumen of the 3D model to inject the culture medium. The experiment was performed at 37°C and 5% CO_2_ to ensure basic cell viability. Owing to the pore structure of hydrogels, the culture medium radially diffused and permeated from the central lumen to the wall of the 3D model. With the perfusion, cells encapsulated in the hydrogels were able to obtain enough nutrients and oxygen and remove metabolic wastes.

**Figure 6:**
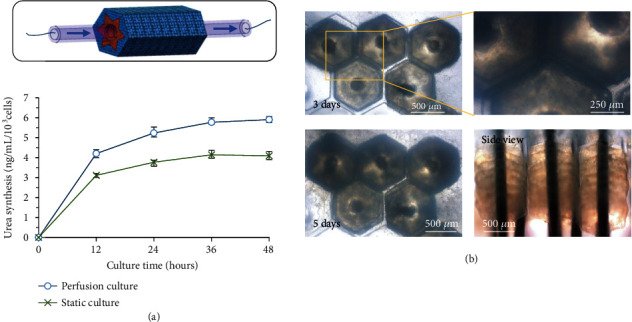
(a) Urea synthesis of the liver lobule-like 3D model during perfusion culture and static culture (mean ± SD, *n* = 3). (b) Scale-up and in vitro culture of the liver lobule-like model.

The urea synthesis of the 3D model during perfusion culture was measured to assess the liver function of the liver lobule-like 3D model. The same 3D model under static culture in the Petri dish was prepared as the control group. The statistical results are shown in Figure 6(a). Urea synthesis was measured in both two groups, which demonstrated that the liver lobule-like 3D model was allowed to synthesize urea during in vitro culture. Both of the groups promoted urea synthesis at the beginning. However, the speed of the urea synthesis of the perfusion group was greater than that of the static group. During the whole culture period, urea synthesis of the perfusion group was significantly greater than that of the static group, demonstrating that the perfusion culture could facilitate the basic function expression of the HepG2 cells in the 3D hydrogel model and the importance of the perfusion ability of the in vitro 3D cellular model.

Owing to the central lumen, the liver lobule-like 3D models can be scaled up with the support of a micropillar array. As shown in Figure 6(b), several liver lobule-like 3D models were integrated and cultured. With incubation at 37°C under 5% CO_2_, cells were successfully grown in the integrated model, indicating the potential of generating in vitro functional liver tissues as drug test models or clinical alternatives using the proposed method.

## 4. Discussion

Engineered liver tissues with in vivo functions are in demand in basic biomedical research and regenerative medicine and for the evaluation of new drugs. Various tissue engineering technologies have been developed to construct hepatocyte culture platforms and liver tissue models. Early-stage two-dimensional (2D) culture systems have achieved the coculture of hepatocytes and nonparenchymal cells to induce cell-cell interactions and enhance cell functions [2, 31]. However, native liver tissue is a 3D culture environment. Cells in a 2D environment lack spatial interactions with surrounding cells and ECM, which may cause loss of normal cell behavior and functions [32, 33]. In contrast, a 3D ECM environment improves the liver-specific functions in hepatocyte cultures compared with conventional 2D cultures [34, 35]. Cell spheroids, cell clusters, and cell sheets have been constructed and used to provide the 3D culture environments of hepatocytes for drug development and testing [36–39]. Ozawa et al. developed an alginate gel-based microwell array for the fabrication of hepatocyte spheroids and coculture with fibroblasts encapsulated in the alginate gel [40]. Yamada et al. proposed a coflowing microfluidic system to produce multilayered hydrogel fibers in which hepatocytes were sandwiched by fibroblasts [41]. However, because of the absence of pores or vessel-like lumens in these structures, rapid nutrients and oxygen transfer of the encapsulated cells cannot be guaranteed. Additionally, liver tissue is a complex organization of multiple cell types with a specific distribution. Ma et al. presented a coculture model with a hexagonal shape and hierarchical cell distribution to mimic native liver module architecture through bioprinting [42]. This model recreates the liver lobule-like hexagonal structure but still lacks 3D spatial cell-cell interactions and is limited to scale up for constructing large-volume complex 3D architectures. To address these limitations, we developed a modular assembly method for hierarchical cell-laden microstructures with heterogeneous patterns into complex 3D cellular models with liver lobule-like morphology and vessel-mimetic lumen. The 3D model with native liver lobule-like morphology can maintain high cell viability during long-term static culture. The 3D model allows perfusion culture through the central lumen for sufficient nutrient and oxygen exchange with encapsulated cells. The urea synthesis with 1.5-fold improvement after perfusion culture demonstrates the liver function of the 3D model and vessel-like significance of the lumen. Furthermore, the 3D models with embedded lumen can be scaled up for culturing large 3D cellular models.

As the assembly units are cell-laden hierarchical microstructures with a liver lobule-like pattern, the assembled 3D model also possesses liver lobule-like architecture and well-defined microregions, where HepG2 cells and HUVECs are encapsulated in the inner PEGDA substructure and outer GelMA substructure, respectively. PEGDA and GelMA are biocompatible hydrogels commonly used in tissue engineering [43, 44]. By photopatterning, PEGDA and GelMA can be cured into an arbitrary pattern with cell encapsulation. Although GelMA mechanical properties can be increased by increasing the GelMA concentration, the cell viability will decrease with high GelMA concentration [45]. Therefore, PEGDA with radial-like morphology is designed as an inner substructure to support the perfusable structure and mimic the radial cell arrangement of the actual liver lobule. Microstructures with arbitrary hierarchical shapes, different cell types, and photosensitive hydrogels can also be fabricated using the multistep photopatterning method. In the liver lobule-like 3D model, although the parenchymal hepatocytes (HepG2) and nonparenchymal cells (HUVECs) are separately encapsulated in different regions, both heterotypic and homotypic cell-cell interactions can be realized. The exchange of soluble factors between HepG2 cells encapsulated in PEGDA and HUVECs encapsulated in GelMA can be achieved because of the intrinsic porous structure of the hydrogels. Additionally, HUVECs can migrate and spread from GelMA to PEGDA during long-term culture, which promotes direct contact of the two kinds of cells.

The well-defined hierarchical 3D model with the involved biodegradable GelMA is beneficial for in vitro cell culture and organization. However, it is challenging to assemble 3D GelMA models since GelMA building blocks are easily damaged and deformed by the driven force. To avoid structural damage during spatial assembly, the pulsed microflow-based on-chip assembly method is proposed for noninvasive 3D assembly of the hierarchical microstructures into a liver lobule-like 3D model. The assembly process is simple, flexible, and efficient. Through fluid force interactions generated by pressing the main capsule of the chip, the microstructures can be spatially moved and stacked layer by layer. The noncontact driven force ensures the structural integrity and fidelity of the hierarchical microstructures, including the outer GelMA substructure. The whole assembly process is performed in a closed liquid environment, which can prevent cell contamination. Microfluidic-based modular assembly methods to construct 3D cellular models through fluid have been reported using force, which most relied on complex microfluidic chips that need to be redesigned and manufactured with customized assembly units [15]. Our proposed on-chip assembly method allows for the assembly of the microstructures with arbitrary shapes and sizes. Moreover, the 3D stacking process is reversible. Once we press the subcapsule, circular flow in the opposite direction can drive upward and push the microstructures on the microneedle to be released one by one. As a result, the layer numbers of the final 3D model are controllable. In summary, the proposed method is advantageous for assembling liver lobule-like 3D models with hierarchical cell distribution and a central lumen, which may provide a useful paradigm for the fabrication of liver alternatives for drug discovery and regenerative medicine.

## Figures and Tables

**Table 1:** The proportion of the streamline distribution in the falling area in different liquid chambers.

Aspect ratio of the liquid chamber	Proportion of the streamline distribution area in the falling area
1 : 1.5	26.822%
1 : 1	34.116%
1.5 : 1	32.944%
